# mTORC1 Is a Local, Postsynaptic Voltage Sensor Regulated by Positive and Negative Feedback Pathways

**DOI:** 10.3389/fncel.2017.00152

**Published:** 2017-05-30

**Authors:** Farr Niere, Kimberly F. Raab-Graham

**Affiliations:** Department of Physiology and Pharmacology, Wake Forest School of MedicineWinston-Salem, NC, United States

**Keywords:** mTOR, syntaxin, glutamate receptors, ion channels, neurological disorders

## Abstract

The mammalian/mechanistic target of rapamycin complex 1 (mTORC1) serves as a regulator of mRNA translation. Recent studies suggest that mTORC1 may also serve as a local, voltage sensor in the postsynaptic region of neurons. Considering biochemical, bioinformatics and imaging data, we hypothesize that the activity state of mTORC1 dynamically regulates local membrane potential by promoting and repressing protein synthesis of select mRNAs. Our hypothesis suggests that mTORC1 uses positive and negative feedback pathways, in a branch-specific manner, to maintain neuronal excitability within an optimal range. In some dendritic branches, mTORC1 activity oscillates between the “On” and “Off” states. We define this as negative feedback. In contrast, positive feedback is defined as the pathway that leads to a prolonged depolarized or hyperpolarized resting membrane potential, whereby mTORC1 activity is constitutively on or off, respectively. We propose that inactivation of mTORC1 increases the expression of voltage-gated potassium alpha (K_v_1.1 and 1.2) and beta (K_v_β2) subunits, ensuring that the membrane resets to its resting membrane potential after experiencing increased synaptic activity. In turn, reduced mTORC1 activity increases the protein expression of syntaxin-1A and promotes the surface expression of the ionotropic glutamate receptor N-methyl-D-aspartate (NMDA)-type subunit 1 (GluN1) that facilitates increased calcium entry to turn mTORC1 back on. Under conditions such as learning and memory, mTORC1 activity is required to be high for longer periods of time. Thus, the arm of the pathway that promotes syntaxin-1A and K_v_1 protein synthesis will be repressed. Moreover, dendritic branches that have low mTORC1 activity with increased K_v_ expression would balance dendrites with constitutively high mTORC1 activity, allowing for the neuron to maintain its overall activity level within an ideal operating range. Finally, such a model suggests that recruitment of more positive feedback dendritic branches within a neuron is likely to lead to neurodegenerative disorders.

## Introduction

The mammalian/mechanistic target of rapamycin (mTOR) is a ubiquitous serine/threonine kinase that is involved in many cellular processes (Hay and Sonenberg, 2004; Zoncu et al., 2011; Laplante and Sabatini, 2012). mTOR forms complexes with two distinct sets of proteins to give rise to mTORC1 and mTORC2, for complex 1 and 2 respectively. mTORC1 is well-characterized for its important roles in nutrient sensing and mRNA translation regulation (Hay and Sonenberg, 2004; Hoeffer and Klann, 2010; Santini and Klann, 2011; Switon et al., 2017). Historically, phosphorylation of mTORC1, which is thought to activate or turn on the protein, is required to signal to its downstream targets to promote mRNA translation or protein synthesis. Recent work, however, is redefining this view by demonstrating that mTORC1 activity is equally important in repressing protein synthesis of select mRNAs (Raab-Graham et al., 2006; Auerbach et al., 2011; Sosanya et al., 2013; Niere et al., 2016). The initial discovery that mTORC1 activity represses the local, dendritic mRNA translation of the voltage-gated potassium channel subunit, K_v_1.1 was pivotal in reexamining mTORC1’s function. In hindsight, the discovery was foretelling mTORC1’s function in membrane excitability. Our subsequent study revealed that acute reduction of mTORC1 activity by rapamycin *in vivo* preferentially alters the expression of proteins involved in ion homeostasis and regulation of the membrane potential (Raab-Graham et al., 2006; Niere et al., 2016). Unlike mTORC1, mTORC2 is insensitive to acute rapamycin treatment (Sarbassov et al., 2006; Lamming et al., 2012; Saxton and Sabatini, 2017). For the purpose of this hypothesis article, we primarily consider mTORC1 regulation of local protein synthesis as an underlying mechanism that alters membrane potential within individual dendrites. To the best of our knowledge, the role of mTORC1 in regulating local, dendritic membrane potential is unexplored. Deciphering the mechanism by which mTORC1 controls the cell’s membrane potential is fundamental, since neurological disorders with aberrant mTORC1 activity present abnormal electrochemical membrane properties (Poolos and Johnston, 2012; Schmunk and Gargus, 2013).

### Local Protein Synthesis in Dendrites

Local mRNA translation is a spatiotemporal mechanism that ensures rapid protein synthesis within a limited region (Steward and Schuman, 2001). Numerous mRNAs have already been identified in neuronal processes; and because of their location, they are poised to be immediately translated in response to activity (Raab-Graham et al., 2006; Cajigas et al., 2012; Niere et al., 2012). In the dendrites, for example, the activity-regulated cytoskeleton-associated (Arc) protein, which facilitates the removal of α-amino-3-hydroxy-5-methyl-isoxazolepropionic acid (AMPA)-selective ionotropic glutamate receptors (GluA), is rapidly synthesized—within 5 min—upon activating group I metabotropic glutamate receptors (mGluRs), resulting in long-term synaptic depression (LTD; Park et al., 2008; Waung et al., 2008; Niere et al., 2012). Additionally, the dendrite-rich region of hippocampal CA1 *stratum radiatum* contains several mRNAs that code for ion channels, bringing to mind that other ion channel proteins, like K_v_1.1, can be locally synthesized upon the right cues, (Table 1; Raab-Graham et al., 2006; Cajigas et al., 2012). The compartmentalization of these mRNAs, away from the soma or axons, suggests that their translation will only alter dendritic membrane properties in a site-specific manner. While the necessary elements (e.g., mRNAs, translation factors and location) to regulate dendritic membrane potential actively are readily available, it still remains unknown whether a general mechanism exists that can coordinate the expression of ion channels, receptors and their associated proteins to change the membrane potential dynamically.

**Table 1:** **List of membrane potential-associated proteins identified by MS/MS in the PSD and soluble fractions of cortices from rats injected with rapamycin or DMSO**.

A. Verified dendritic mRNAs	PSD	Soluble
ATPase Na^+^/K^+^ transporting subunit alpha 1 (Atp1a1)	–	On
ATPase Na^+^/K^+^ transporting subunit alpha 3 (Atp1a3)	–	–
ATPase sarcoplasmic/endoplasmic reticulum Ca^2+^ transporting 2 (Atp2a2)	On	Off
Calcium voltage-gated channel auxiliary subunit alpha2delta 1 (Cacna2d1)	Off	ND
Contactin associated protein 1 (Cntnap1)	On	ND
Dipeptidyl peptidase like 6 (Dpp6)	On	Off
Discs large MAGUK scaffold protein 1 (Dlg1)	ND	Off
Discs large MAGUK scaffold protein 4 (Dlg4)	On	ND
Drebrin 1 (Dbn1)	On	ND
G protein subunit alpha q (Gnaq)	–	Off
Glutamate ionotropic receptor NMDA type subunit 1 (Grin1)	Off/OR	ND
Guanine nucleotide binding protein, alpha 11 (Gna11)	Off	On
Neurexin 1 (Nrxn1)	ND	Off/OR
Neuroligin 2 (Nlgn2)	ND	–
Neuroligin 3 (Nlgn3)	ND	Off
Potassium voltage-gated channel subfamily A member 1 (Kcna1)	ND	Off
Potassium voltage-gated channel subfamily A regulatory beta subunit 2	Off	ND
Solute carrier family 17 member 7 (Slc17a7)	On	Off
Syntaxin 1A (Stx1a)	Off	–
Syntaxin 1B (Stx1b)	On	On
Synuclein alpha (Snca)	ND	Off
Valosin-containing protein (Vcp)	On	–
**B. Unverified dendritic mRNAs**		
4-aminobutyrate aminotransferase (Abat)	–	Off
ATPase Na^+^/K^+^ transporting subunit beta 1 (Atp1b1)	–	On
ATPase Na^+^/K^+^ transporting subunit beta 2 (Atp1b2)	ND	Off
ATPase Na^+^/K^+^ transporting subunit beta 3 (Atp1b3)	On	–
Calcium/calmodulin-dependent protein kinase type II subunit delta (Camk2d)	–	Off
Cyclin-dependent kinase 5 (Cdk5)	ND	Off
Dihydrolipoamide dehydrogenase (Dld)	–	–
EH-domain containing 3 (Ehd3)	Off	On
Gap junction protein, alpha 1 (Gja1)	On	ND
Kinesin family member 5B (Kif5b)	ND	Off/OR
NADH dehydrogenase (ubiquinone) Fe-S protein 1 (Ndufs1)	–	–
Parkinsonism associated deglycase (Park7)	On	On
Peroxiredoxin 3 (Prdx3)	ND	Off
Protein kinase C, epsilon (Prkce)	ND	Off
Protein phosphatase 3 catalytic subunit alpha (Ppp3ca)	On	–
Protein phosphatase 3 regulatory subunit B alpha (Ppp3r1)	Off	
Stomatin like 2 (Stoml2)	Off	On/OR
Superoxide dismutase 1, soluble (Sod1)	Off	ND
Tyrosine 3-monooxygenase/tryptophan 5-monooxygenase activation protein, epsilon (Ywhae)	–	On
Tyrosine 3-monooxygenase/tryptophan 5-monooxygenase activation protein, eta (Ywhah)	Off	On
WD repeat domain 1 (Wdr1)	On	Off

*The list was generated using DAVID to identify proteins that belong to “GOTERM BP ALL—Regulation of membrane potential”. (A) List of proteins whose mRNAs have been verified to reside in the dendrite as reported (Cajigas et al., 2012). (B) List of proteins whose mRNAs have not been verified to reside in the dendrite. “On” indicates that protein level was reduced in PSD or soluble when mTOR was inhibited by rapamycin as described (Niere et al., 2016). “Off” denotes that the protein level was elevated when mTOR was inhibited. “–” indicates that protein expression did not change when mTOR activity was perturbed. “ND” signifies that the protein was not detected in the fraction by MS/MS. “OR” denotes that the protein was out-of-range, such that it could only be detected when mTOR was active (On/OR) or inhibited (Off/OR)*.

### mTORC1-Regulated Protein Synthesis and Plasticity

Engagement of receptor-mediated signaling such as glutamate, brain-derived neurotrophic factor (BDNF), and γ-amino butyric acid (GABA) receptors under distinct neuronal conditions alters membrane potential and involves mTORC1 (Hou and Klann, 2004; Takei et al., 2004; Inamura et al., 2005; Bateup et al., 2011; Weston et al., 2012, 2014; Workman et al., 2013, 2015). Our recent finding that acute inhibition of mTORC1 activity disrupts the expression of proteins that are involved in ion homeostasis and membrane potential at the synapse supports the close relationship of mTORC1 signaling in maintaining a normal electrochemical gradient of the postsynaptic membrane (Niere et al., 2016). Using the unbiased approach of tandem mass spectrometry (MS/MS) to identify changes in protein composition at the postsynaptic site, we found proteins whose functions affect the membrane potential (Table 1). Several of the mRNAs that encode these proteins curiously reside in the dendrites, suggesting that membrane potential-associated proteins can be locally translated with the right cues (Raab-Graham et al., 2006; Cajigas et al., 2012). We also identified proteins whose levels increased (mTORC1-Off), decreased (mTORC1-On) or remained consistent (mTORC1-independent) with mTORC1 inhibition (Niere et al., 2016).

### Dynamic Expression of Dendritic Voltage-Gated Potassium Channel

A family of ion channels that significantly impact the membrane potential is made up of voltage-gated potassium channels (K_v_). (Magee and Johnston, 2005; Metz et al., 2007; Pongs, 2008; Remy et al., 2010). While we have a good grasp on the mechanics of K_v_ channels and their influence on the electrical properties of the cell membrane, little is known about posttranscriptional mechanisms that allow them to respond accordingly to changes in their extracellular environment. The members of the subfamily of K_v_1 channels are of particular interest, as their presence or absence can profoundly affect the resting membrane potential and the generation of action potentials (Rho et al., 1999; Brew et al., 2003; Gittelman and Tempel, 2006; Kirchheim et al., 2013; Sosanya et al., 2015a). K_v_1 channels form octomers that consist of four pore-forming α subunits (K_v_α1.X) and four cytoplasmic β (K_v_β) subunits (Trimmer, 1998; Gutman et al., 2005). The α subunit K_v_1.1 is profoundly vital as majority of disease-associated mutations in K_v_1 are in the *KCNA* gene, which codes for K_v_1.1 (Ovsepian et al., 2016). Because of the inherent fluid structure of the membrane, ion channels are inserted into and removed from the membrane (Hoffman et al., 1997; Yuste, 1997). The proper surface expression of K_v_1.1-containing channels depends on the co-assembly of K_v_1.1 with other K_v_α1-type (e.g., K_v_1.2, K_v_1.3 or K_v_1.4) channels to mask the strong endoplasmic reticulum (ER) retention signal of K_v_1.1 (Vacher et al., 2007). In the dendrites, inhibition of mTORC1 promotes new synthesis of K_v_1.1 protein and its expression on the membrane (Raab-Graham et al., 2006). With the identification of K_v_1.1 and in anticipation of identifying other “K_v_1.1-like” proteins whose levels are negatively regulated by mTORC1 activity, we have designated K_v_1.1 and “K_v_1.1-like” proteins as “mTORC1-Off”, since they require mTORC1 activity to be turned off or inhibited to undergo mRNA translation. Conversely, “mTORC1-On” proteins require turning on or activation of mTORC1 to be synthesized.

### Dysregulated Protein Synthesis Underlies Neurological Disorders

Healthy cells require mTORC1 activity to be within a dynamic range, since neurons in static mTORC1 states (i.e., over- or underactive) are linked to several neurological disorders (Pei and Hugon, 2008; Hoeffer and Klann, 2010; Ma et al., 2010; Bové et al., 2011; Jernigan et al., 2011; Ricciardi et al., 2011; Santini and Klann, 2011; Costa-Mattioli and Monteggia, 2013; Switon et al., 2017). Disorders with excessive protein synthesis are generally associated with overactive mTORC1, while reduced protein production with underactive mTORC1. But in light of the discovery that mRNA translation occurs both when mTORC1 is turned on or off, a state of fixed mTORC1 activity can conceivably promote and repress the protein synthesis of dendritically localized mRNAs to pathological levels. A prime example of a neurological disorder with hyperactive mTORC1 is temporal lobe epilepsy (TLE; Buckmaster et al., 2009; Zeng et al., 2009; Pun et al., 2012; Sha et al., 2012; Wong, 2014; Sosanya et al., 2015a). It has been suggested that overactive mTORC1 in epilepsy promotes excessive protein synthesis that engenders neuronal hyperexcitability. Equally as important, overactive mTORC1 represses several ion channels that reduces excitability. This arm of active mTORC1 also supports epilepsy by giving rise to aberrant synchronous activity of neurons and neuronal circuits (Raab-Graham et al., 2006; Graef and Godwin, 2010; Poolos and Johnston, 2012; Brewster et al., 2013; Sosanya et al., 2013, 2015a; Niere et al., 2016). The imbalance caused by increased synthesis of mTORC1-On proteins and decreased production of mTORC1-Off proteins thrusts the neuronal activity outside the optimal operating range, consequently bringing forth devastating neurological disorders.

### mTORC1 as a Local Voltage Sensor

mTORC1 controls mRNA translation of ion channels, ionotropic receptors and their associated proteins (Gong et al., 2006; Raab-Graham et al., 2006; Antion et al., 2008; Bateup et al., 2011, 2013; Huang et al., 2012; Weston et al., 2012; Sosanya et al., 2013, 2015a). In light of these findings and our recent discoveries, we hypothesize that mTORC1 serves as a local voltage sensor by coordinating the levels of postsynaptic proteins that regulate the membrane’s electrical property. We propose below that mTORC1 employs negative and positive feedback mechanisms to modify the electrical features of the dendritic membrane.

## Materials and Methods

### Sample Preparation

Sprague Dawley rats and C57BL/6 mice (4–6 weeks old) were used. Rapamycin was administered intraperitoneally at 10 mg/kg into rats and 1 mg/kg into mice. As control, animals received an equal volume and concentration of DMSO, which was the vehicle for rapamycin. Synaptoneurosomes were prepared from cortices by size filtration (Workman et al., 2013). The postsynaptic density fraction was isolated as described (Niere et al., 2016). Briefly, synaptoneurosomes were subjected to Triton X-100 solubilization. The PSD and soluble fractions constituted the Triton X-100 insoluble and soluble portions, respectively.

### Western Blots

Total lysates, synaptoneurosomes and PSD were solubilized in RIPA buffer before running SDS-PAGE. Protein concentrations were measured using BCA protein assay. Equal amount of protein from each sample was loaded and run in SDS-PAGE gel. Proteins were immunoblotted with primary antibodies against phospho-mTOR Ser2448 (1:2000; Cell Signaling), mTOR (1:5000; LifeTechnologies), K_v_1.1 (1:1000 NeuroMab), K_v_1.1 (1:1000; LifeSpan BioSciences), K_v_1.2 (NeuroMab), K_v_β2 (NeuroMab), actin (1:10,000; Sigma), tubulin (1:50,000; Abcam). Immunoblots were washed and incubated with the appropriate fluorescence- (LiCor) or HRP-conjugated (ThermoFisher Scientific) secondary antibodies (1:5000). Protein expression was quantified by densitometric analysis using ImageJ (National Institutes of Health) software. For the PSD fraction, total protein as measured by densitometry of Ponceau-S staining was used to normalize K_v_1.1 and K_v_1.2 protein expression.

### Immunoprecipitation

Rat cortices were homogenized in tris-buffered saline (50 mM). Membrane fraction was sedimented at 14,000 RPM for 20 min at 4°C. Isolated membranes were solubilized (20 mM HEPES, pH 7.2 150 mM NaCl, 5 mM EDTA, 1% Triton X-100, protease inhibitor, 0.5% SDS) to extract membrane proteins. Preclearing and immunoprecipitation were performed with Protein A/G-agarose suspension (Roche) using the manufacturer’s directions. 4 μg of rabbit-anti-K_v_1.1 (LifeSpan Biosciences) or rabbit IgG (control, Santa Cruz Biotechnologies) were used to precipitate the K_v_1.1 protein complex from the pre-cleared samples (1 mg of protein). Laemmli sample buffer (1×, BioRad) was used to dissociate the antibody-antigen complex from the agarose beads, after which the supernatant was run in SDS-polyacrylamide gel by electrophoresis. To probe for K_v_1.2 protein, mouse-anti-K_v_1.2 (NeuroMab) was used. Immunoblots were visualized with the appropriate fluorescence-conjugated secondary antibodies (1:5000; LiCor).

### Immunofluorescence

Primary hippocampal rat neurons were prepared from postnatal rats (day 0–2) and fixed in PFA at room temperature as described (Niere et al., 2012; Workman et al., 2013). The following primary antibodies were used: rabbit-anti-K_v_1.1 (1:200; LifeSpan Biosciences), mouse-anti-K_v_1.2 (1:200; NeuroMab), and chicken-anti-MAP2 (1:2000; Aves Labs). Appropriate secondary antibodies—AlexaFluor-488, 555 and 647 (Invitrogen)—were used to visualize the proteins of interest. K_v_1.1, K_v_1.2 and MAP2 protein expression were quantified in secondary dendrites that were 50 μm in length and at least 20 μm away from the soma as described (Niere et al., 2012, 2016). A Pearson’s correlation coefficient (PCC) was determined to measure K_v_1.1–1.2 colocalization in the dendrites as described (Niere et al., 2016). Z-stacks of seven planes at 1 μm/plane were acquired for each dendrite. Only the plane that had the brightest MAP2 staining was analyzed for colocalization. The ImageJ (NIH) plugin JACoP was used to determine the PCC for each dendrite. The same threshold paradigm was used across all conditions using the threshold function of JACoP.

## Biochemical and Bioinformatics Analyses of Neuronal Proteome Expose An mTORC1-Mediated Regulation of Local, Postsynaptic Excitability

**Figure 1:**
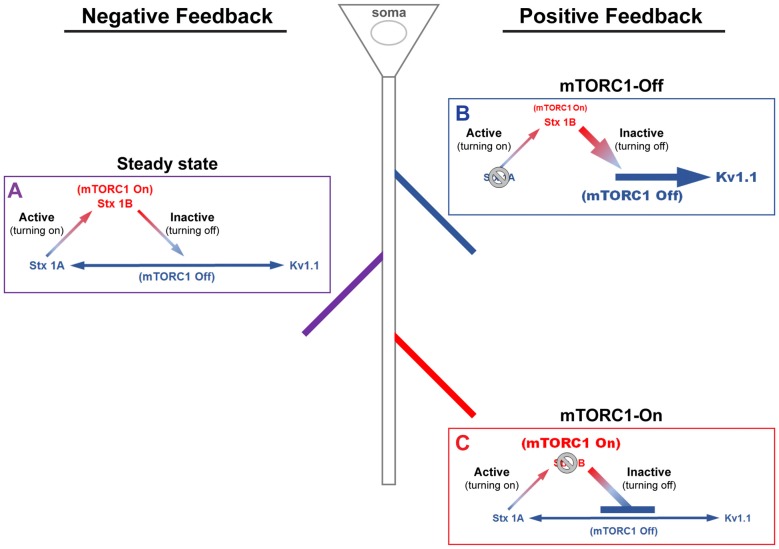
**A model of mammalian/mechanistic target of rapamycin complex 1 (mTORC1)-mediated regulation of postsynaptic membrane excitability**. mTORC1 regulates membrane excitability by coordinating the expression of mTORC1-On and Off proteins. **(A)** At steady-state, mTORC1 dynamically switches between On and Off forms in response to neuronal activity. Turning on mTORC1 (Active; ascending arrow) increases the level of syntaxin 1B (Stx1B), an mTORC1-On protein that promotes endocytosis of N-methyl-D-aspartate (NMDA) receptors (NMDARs). Removal of NMDARs reduces membrane excitability and turns off mTORC1 activity (Inactive; descending arrow). Turning off mTORC1 increases the expression of mTORC1-Off proteins, syntaxin 1A (Stx1A; *bottom, left arrow*) and voltage-gated potassium channel (K_v_1.1, *bottom, right arrow*). Stx1A shuttles NMDARs to the membrane surface, which turns on mTORC1 and increases membrane excitability. K_v_1.1, on the other hand, dampens synaptic input. The number of activated NMDARs at the surface, which turns on mTORC1, acts as a signal to stop K_v_1.1 mRNA translation. **(B)** In the absence of Stx1A (or *Stx1a* mRNA), a positive feedback mechanism could be triggered, whereby mTORC1 remains turned off. The inability to reinsert NMDARs would further lower the membrane potential and support K_v_1.1 protein synthesis, reducing the threshold for synaptic activation. **(C)** The absence of Stx1B (or *Stx1b* mRNA) could initiate another positive feedback mechanism, such that mTORC1 activity is constitutively on and the membrane remains potentiated. In postsynaptic regions that lack Stx1B, suppression of NMDAR exocytosis would cause mTORC1 to stay active and lower K_v_1.1 expression. This state may be another mechanism that supports long-term potentiation.

We have previously shown that stimulation of the ionotropic glutamate receptor N-methyl-D-aspartate (NMDA)-type (GluN or NMDAR) led to downstream activation of mTORC1 that can be blocked by (2 R)-amino-5-phosphonovaleric acid (AP5)—NMDAR antagonist—and rapamycin (Raab-Graham et al., 2006; Sosanya et al., 2013, 2015a). Recently, our MS/MS data suggest that mTORC1 regulates the expression of three proteins that can cause dynamic changes in membrane excitability: syntaxin 1A (Stx1A), syntaxin 1B (Stx1B), and K_v_1.1. These proteins work together to engage negative and positive feedback pathways that control mTORC1 activity (Figure 1). The negative feedback back loop has two arms to turn mTORC1 on or off. Turning off mTORC1 activity promotes the expression of Stx1A, an mTORC1-Off protein. Stx1A serves to switch mTORC1 activity from an “Off” to an “On” state by increasing the insertion of NMDARs to the extracellular membrane. With the increase in mTORC1 activity, the mTORC1-On protein, Stx1B, is then synthesized. Stx1B causes the endocytosis of NMDARs, thus promoting the transition of mTORC1 from the “On” to “Off” state (Figure 1). Notably, when mTORC1 is on, then the translation of the positive feedback mRNAs (i.e., K_v_1.1, K_v_1.2, K_v_β2) are repressed. In contrast, reduction of mTORC1 activity also enhances K_v_1.1, K_v_1.2, and K_v_β2 protein levels that work to sustain the mTORC1-Off state. Whether a dendritic branch utilizes positive or negative feedback is likely to depend on the presence or absence of Stx1A and/or Stx1B within a dendritic branch (Figure 1).

## mTORC1-Mediated Positive Feedback Regulation of Local, Dendritic Membrane Potential Through K_v_1.1 Protein Synthesis

Potassium channels are likened to “shock absorbers” as they dampen dendritic membrane depolarization that can arise from calcium entry (Hoffman et al., 1997; Yuste, 1997; Yuan and Chen, 2006). Inhibiting the activity of mTORC1, interestingly, elevates K_v_1.1 protein expression only in the dendrites (Raab-Graham et al., 2006; Sosanya et al., 2013, 2015a). While we have determined the molecular mechanism that describes local K_v_1.1 protein synthesis, the functional significance of increased K_v_1.1 protein when mTORC1 activity is reduced is not fully understood (Sosanya et al., 2013, 2015a). We propose that the mTORC1-Off-dependent translation of K_v_1.1 serves to ensure that the membrane resets to a normal resting potential, thereby maintaining neuronal excitability to be within an optimal operating range.

### Increased K_v_1.2 and K_v_β2 Protein Levels in mTORC1-Off State May Lead to Increased Postsynaptic Surface Expression of K_v_1.1

K_v_1.1 is an obligate heteromultimeric channel in mammalian neurons (Manganas and Trimmer, 2000). mTORC1 inhibition promotes total and surface expression of dendritic K_v_1.1 that are open at rest (Hopkins et al., 1994; Smart et al., 1998; Brew et al., 2003; Raab-Graham et al., 2006; Sosanya et al., 2013, 2015a). In rapamycin, which reduces mTORC1 activity globally, elevated expression of K_v_1.1 increases the threshold for action potential firing (Sosanya et al., 2015a). However, the proteins that assemble with dendritic K_v_1.1 to facilitate its surface expression are unknown. Two promising candidate proteins identified by our MS/MS that can facilitate the surface expression of K_v_1.1 are K_v_1.2 and β2 (Manganas and Trimmer, 2000). Thus, we set out to determine if K_v_1.2 or β2 subunits are synthesized and co-assemble with K_v_1.1 when mTORC1 activity is reduced.

**Figure 2:**
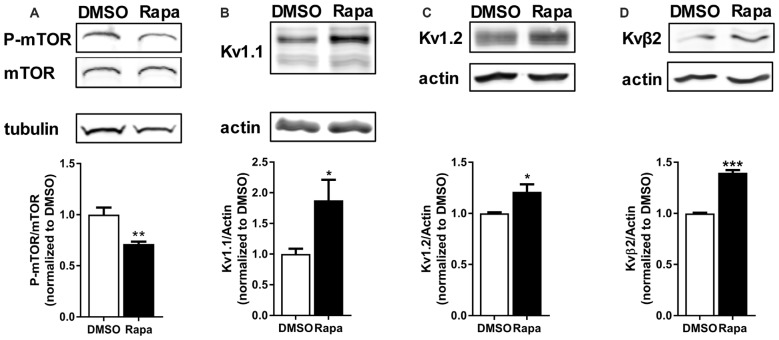
**Reduction of mTOR activity increased protein levels of potassium channels and their associated protein**. Intraperitoneal (i.p.,) administration of rapamycin (Rapa; 1 mg/kg; 1 h), an mTOR inhibitor, in mice **(A)** reduced mTOR activity (DMSO = 1.00 ± 0.07; Rapa = 0.71 ± 0.02), but increased **(B)** K_v_1.1 (DMSO = 1.00 ± 0.09; Rapa = 1.88 ± 0.33), **(C)** K_v_1.2 (DMSO = 1.00 ± 0.01; Rapa = 1.21 ± 0.07), and **(D)** K_v_β2 (DMSO = 1.00 ± 0.01; Rapa = 1.40 ± 0.03) as measured by densitometry analysis. P-mTOR and mTOR denote phosphorylated mTOR and total mTOR, respectively. Representative images and quantification (average ± SEM) are shown. *N* = 4 (DMSO) and 3 (Rapa) animals. Statistics: Student’s *t*-test; *p < 0.05, **p < 0.01, ***p < 0.001.

We first compared K_v_1.2 and β2 expression of cortical total membrane fractions isolated from rats that received an intraperitoneal (i.p.,) injection of carrier (DMSO) or the mTORC1 inhibitor rapamycin. To ensure that rapamycin reduced mTOR activity we quantified the phosphorylated or active form of mTOR normalized to total mTOR by Western blot analysis. Rapamycin reduced mTOR activity by ~30% (Figure 2A). Notably, K_v_1.1, 1.2 and β2 protein levels increased by ~87%, 20% and 40%, respectively, with mTORC1 inhibition (Figures 2B–D).

**Figure 3:**
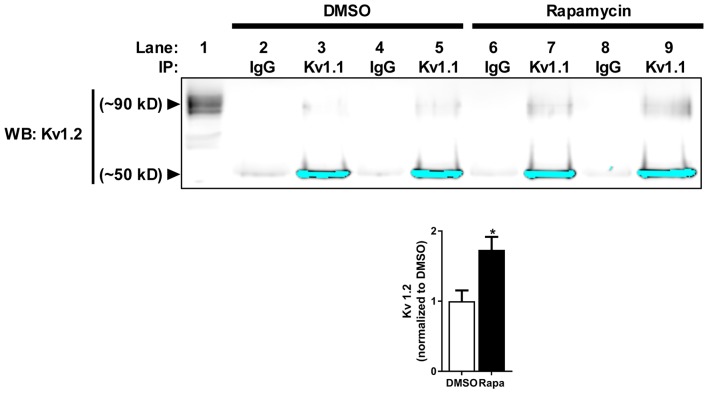
**Reduction of mTOR activity increases K_v_1.1–1.2 association *in vivo***. K_v_1.2 subunit co-immunoprecipitates with K_v_1.1. The high molecular weight (~90 kD) K_v_1.2 subunit assembles more with K_v_1.1 in mice treated with rapamycin (1.73 ± 0.19) compared to DMSO (1.00 ± 0.15) as measured by densitomery analysis. IP denotes the antibody used to immunoprecipitate K_v_1.1 from samples (1 mg/mL) in lanes 2–9. IgG serves as antibody control for immunoprecipiration (lanes 2, 4, 6 and 8). K_v_1.2 is enriched in samples that contain K_v_1.1 antibody-conjugated beads (lanes 3, 5, 7 and 9) compared to IgG-conjugated beads. The signals at ~50 kD are the low molecular K_v_1.2 species and have been saturated to visualize K_v_1.2 at ~90 kD. The high molecular weight is the extracellular fraction, and the low molecular is the intracellular fraction of K_v_1.2 (Zhu et al., 2003). Lane 1 is the input lane, which is 1% (10 μL) of the total volume that was used to immunoprecipitate K_v_1.1. Representative images and quantification (average ± SEM) are shown. *N* = 4 animals per condition. Statistics: Student’s *t*-test; *p < 0.05.

Because mTORC1 inhibition led to a small but significant increase in K_v_1.2 expression, we predicted that the number of K_v_1.2 subunits that associate with new K_v_1.1 channel protein might increase. To test this hypothesis, we immunoprecipitated K_v_1.1 from cortical membranes isolated from mice treated with carrier (DMSO) or rapamycin and Western blotted for K_v_1.2. Indeed, the amount of surface-expressed K_v_1.2 (~90 kD) that co-immunoprecipitated with K_v_1.1 in rapamycin-treated samples increased by ~72% (Figure 3; Zhu et al., 2003). These data altogether suggest that more K_v_1.1 subunits co-assemble with K_v_1.2 with mTORC1 inhibition.

**Figure 4:**
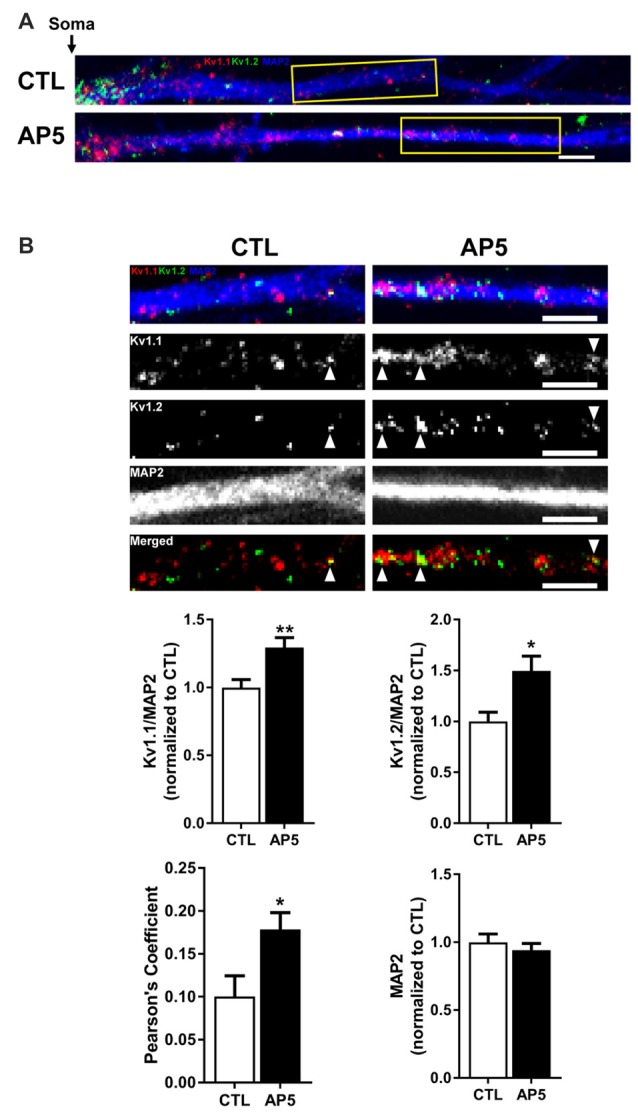
**NMDA receptor blockade by AP5 elevates K_v_1.1 and K_v_1.2 protein in dendrites. (A)** Representative dendrites starting from the edge of the soma (indicated by the black arrow) of dissociated hippocampal neurons treated with (*top*) water (CTL; control) or (*bottom*) AP5, an NMDA receptor blocker. The dendrites were immunostained with antibodies against K_v_1.1 (red), K_v_1.2 (green) and the dendritic marker MAP2 (blue). Dendrites outlined in yellow are magnified in **(B)**. (**B,** *Top*) K_v_1.1, K_v_1.2 and MAP2 expression were measured in distal dendrites (at least 20 μm from the soma). White arrowhead indicates colocalized K_v_1.1 and K_v_1.2 signals. The bottom panel of dendrites show the merged K_v_1.1 and K_v_1.2 immunostaining. Colocalized K_v_1.1 and K_v_1.2 signals appear yellow and are quantified by Pearson’s Coefficient. (*Below*) Quantification (average ± SEM) of fluorescent signal normalized by MAP2 signal indicating more K_v_1.1 and K_v_1.2 in NMDA receptor blocker AP5 compared to control (CTL). K_v_1.1 (CTL = 1.00 ± 0.06, *N* = 24 dendrites; AP5 = 1.29 ± 0.07, *N* = 30 dendrites). K_v_1.2 (CTL = 1.00 ± 0.09, *N* = 11 dendrites; AP5 = 1.50 ± 0.15, *N* = 16 dendrites). MAP2 (CTL = 1.00 ± 0.06; AP5 = 0.94 ± 0.05). NMDA receptor blockade increases colocalization of K_v_1.1 and K_v_1.2 proteins (CTL = 0.10 ± 0.02, *N* = 18 dendrites; AP5 = 0.18 ± 0.02, *N* = 21 dendrites) Statistics: Student’s *t*-test; *p < 0.05, **p < 0.01.

To determine if the increase in K_v_1.1–1.2 heteromultimeric channels with mTORC1 inhibition takes place in the dendrites, we performed imaging experiments that double-labeled K_v_1.1 and K_v_1.2 in cultured hippocampal neurons. As mentioned above, we previously showed that NMDAR stimulation led to downstream mTOR activation (Raab-Graham et al., 2006; Sosanya et al., 2013, 2015a). We initiated these studies by first determining whether K_v_1.1 and K_v_1.2 levels increased in the dendrites of neurons that were treated with the NMDAR inhibitor AP5 relative to carrier (H_2_O; Figures 4A–B). Indeed, both K_v_1.1 and K_v_1.2 expression significantly increased in the dendrites of hippocampal neurons with mTORC1 inhibition. Microtubule-associated protein (MAP2), a dendritic marker, remained constant between treatments (Figure 4B). Consistent with our Western blot results, blocking NMDAR/mTOR signaling increased the dendritic expression of K_v_1.1 and K_v_1.2 (Figures 3, 4). We also examined if we could detect increased colocalization between K_v_1.1 and K_v_1.2 in the dendrites, since our co-immunoprecipitation (coIP) data indicated elevated K_v_1.1–1.2 association when mTORC1 activity is attenuated (Figure 3). Corroborating our coIP data, we detected more K_v_1.1–1.2 colocalization in the dendrites treated with AP5 by Pearson’s correlation analysis (CTL = 0.10 ± 0.02; AP5 = 0.18 ± 0.02; Figure 4B).

**Figure 5:**
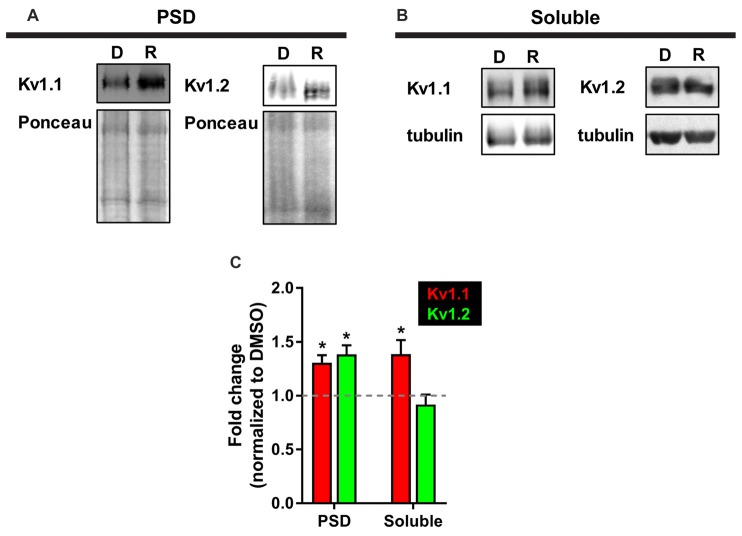
**More K_v_1.1 and K_v_1.2 cofractionate in the PSD with mTORC1 inhibition *in vivo*. (A)** Representative Western blots depicting increased levels of K_v_1.1 and K_v_1.2 in the PSD fraction (Triton X-100 insoluble) of synaptoneurosomes with mTORC1 inhibition by rapamycin (R). DMSO (D) served as control. Total protein as measured by densitometry of Ponceau-S staining was used to normalize K_v_1.1 and K_v_1.2 protein expression. **(B)** In the soluble fraction (Triton X-100 soluble) of synaptoneurosomes, only K_v_1.1 protein was increased with rapamycin. Tubulin was used to normalize K_v_1.1 and K_v_1.2 protein levels. **(C)** Quantification (average ± SEM) is expressed as densitometric ratio between protein values in rapamycin-treated and DMSO-treated rats. K_v_1.1 (PSD = 1.31 ± 0.07; Soluble = 1.39 ± 0.13). K_v_1.2 (PSD = 1.39 ± 0.08; Soluble = 0.92 ± 0.09). *N* = 3 animals per condition. Statistics: Student’s *t*-test; *p < 0.05.

While we observed elevated levels of K_v_1.1 and K_v_1.2 proteins at the dendrites, we were curious whether these changes extended to the PSD. We utilized a biochemical approach that isolates neuronal subcellular fractions using filtration and detergent solubility to examine K_v_1.1 and K_v_1.2 expression in a PSD-enriched fraction. It is well established that resident postsynaptic density (PSD) proteins are not soluble in the detergent Triton X-100 (Fiszer and Robertis, 1967). Thus, we reasoned that we could determine K_v_1.1 and 1.2 by biochemically isolating the lysate (total), Triton X-soluble (dendrites and axons), and Triton X-insoluble (PSD) as outlined in Figure 2 of Niere et al. (2016), (Fiszer and Robertis, 1967; Cohen et al., 1977; Rao and Steward, 1991; Villasana et al., 2006). Summarily, we first assessed the purity of fractionated samples by Western blotting for well-characterized resident proteins: postsynaptic density protein of 95 kD (PSD95, PSD marker) and synapsin 1 (presynaptic marker). The PSD (P) fraction was enriched for PSD95, while little synapsin was detected. The soluble (S) fraction, which mainly contains dendrites and axons, was enriched in synapsin and devoid of PSD95. To ensure that mTORC1 activity was inhibited by rapamycin in the PSD, we probed for the phosphorylated or active form of ribosomal S6 protein (P-S6), the downstream marker of mTORC1, and normalized P-S6 over total S6 protein. As expected, rapamycin reduced P-S6 signal by ~88% (Niere et al., 2016). Western blot analysis for K_v_1.1 and K_v_1.2 in these fractions indicated that K_v_1.1 increased in both the PSD and the soluble fractions, while K_v_1.2 only increased in the PSD with rapamycin (Figure 5). Control proteins, PSD95 and tubulin, did not significantly change between treatments. These data suggest that K_v_1.1–1.2 heteromultimers are likely to reside in the PSD when mTORC1 activity is low.

### Leucine-Rich Glioma Inactivated-1 (Lgi1) Protein and K_v_1.1 May Sustain mTORC1-Off State and Reduced Membrane Excitability Postsynaptically

Our neuronal proteome analysis of different cellular compartments detected three proteins known to interact with K_v_1.1 channels that are regulated by mTORC1 activity. Of particular interest is the highly prominent epilepsy-associated protein Lgi1 (Anderson, 2010; Yokoi et al., 2012; Kegel et al., 2013; Ohkawa et al., 2013). PSD (P) and soluble (S) fractions composed of axons and dendrites—were derived from synaptoneurosomes and subjected to Triton X-100 solubilization. Rapamycin increased the expression of Lgi1 in the Triton X-100 insoluble, PSD (P) fraction, while reducing it in the Triton X-100 soluble (S) fraction—(*P* = 129%, *S* = 65% of control; Niere et al., 2016). Interestingly, *Lgi1* null mice exhibit neuronal hyperexcitability and spontaneous seizures and fail to live beyond 25 days (Chabrol et al., 2010; Fukata et al., 2010; Yu et al., 2010). Lgi1 is viewed as an antiepileptogenic protein as it converts K_v_1.1 channels from A-type channels that inactivate quickly to a delayed rectifier by preventing K_v_1.1–K_v_β1 interaction (Chernova et al., 1998; Fukata et al., 2006, 2010; Schulte et al., 2006). Thus, the increased postsynaptic expression of Lgi1 could further enhance the conductance of K_v_1.1 in the PSD and in turn keep mTORC1 in a dephosphorylated state. In addition, the reduced expression in the soluble fraction suggests that K_v_1.1 expression in the dendrites and/or axons may associate with K_v_β1 and quickly inactivate, similar to A-type channels.

### Bidirectional Regulation of Cyclin-Dependent Kinase 5 (Cdk 5) May Support mTORC1-Off-Dependent Reduction of Postsynaptic Membrane Excitability

We found that Cdk5 was differentially regulated in soluble and PSD fractions (Table 1). mTORC1 inhibition increased Cdk5 protein expression in the soluble. Phosphorylation of K_v_β2 by Cdk2/5 had been shown to disrupt K_v_β2’s binding to EB1, thus releasing the channel complex from microtubules and allowing K_v_1.1-complex to traffic to the plasma membrane (Gu et al., 2006; Vacher and Trimmer, 2011). The increased level of Cdk5, therefore, may facilitate the local insertion of K_v_1.1-containing channels in the dendrites when mTORC1 is inhibited. In the PSD, however, we did not detect Cdk5 (Table 1) but found K_v_1.1 and K_v_1.2 (Figure 5). These findings suggest that while Cdk5 may be essential for the trafficking of K_v_1.1 channels to the dendritic surface, this function of Cdk5 may not be critical in the PSD. The absence of microtubules in spines also support the notion that Cdk5 is not necessary to traffic the K_v_1.1 complex to the membrane of the PSD compartment. The masking of the ER retention signal of K_v_1.1 by associating with K_v_1.2 in the PSD may be sufficient to traffic the K_v_1.1 complex to the membrane surface (Manganas and Trimmer, 2000; Tiffany et al., 2000).

### Potential Physiological Impact of Compartmentalized mTORC1 Activity

These data collectively suggest that mTORC1 may determine the subcellular localization of K_v_1.1-associated proteins and K_v_1.1-containing channels. Localized K_v_1.1 complex, in turn, will dictate the electrical properties of the compartment and whether multiple compartments (i.e., spines and/or dendritic branches) can interact. For example, potassium channels have been suggested to serve as gate keepers, electrically isolating neuronal compartments (Metz et al., 2007; Harnett et al., 2013). When mTORC1 activity is low in the PSD, the expression of K_v_1.1, 1.2, and Lgi1 (sustained potassium current) is likely to temper synaptic stimulation by preventing the opening of voltage-dependent calcium channels and thus confining the synaptic signal to the stimulated spine itself (Harnett et al., 2012). In a situation where mTORC1 activity is high in the spine but low in the associated dendritic branch, reduced Lgi1 would suggest that K_v_1.1 could associate with K_v_β1 (transient or A-type channel). K_v_1.1-β1 would quickly inactivate upon depolarization allowing for the interaction of nearby stimulated spines (Harnett et al., 2013). In this case, dendritic branches (daughter branches) are likely to remain uncoupled from their parent dendrite. When mTORC1 activity is high throughout the dendritic branch, then K_v_ expression is repressed. As a consequence, synaptic stimulation will generate local calcium and/or sodium spikes that invade into the parent dendrite (Golding et al., 1999; Frick et al., 2003; Losonczy et al., 2008; Makara et al., 2009). mTORC1 activity, therefore, serves as a local voltage sensor, with increased activity reducing barriers (K_v_ channels) between compartments allowing for dendritic integration.

## mTORC1-Mediated Negative Feedback Regulation of The Postsynaptic Membrane Excitability Through The Differential Expression of Stx1A, Stx1B, and GluN1 Proteins

A family of proteins that stood out in our MS/MS analysis of the proteome that localize with the PSD was syntaxin 1 (Niere et al., 2016). We discovered that mTORC1 regulated syntaxin-1 protein levels and that the two isoforms, Stx1A and Stx1B, differentially responded to mTORC1 activity level (Table 1). Attenuating mTORC1 activity with the mTOR specific inhibitor rapamycin (10 mg/kg, 1 h) increased Stx1A expression (~54%) but reduced Stx1B (~24%; Niere et al., 2016). Therefore, we classified Stx1A as mTORC1-Off and Stx1B as mTORC1-On. Syntaxin-1 is well-characterized as a presynaptic protein that regulates neurotransmitter release; however, its function postsynaptically remains unclear (Bennett et al., 1992, 1993; Rizo and Südhof, 2002, 2012; Rizo and Xu, 2015). Contrary to earlier assumptions, recent studies now reveal that presynaptic syntaxin-1A and 1B perform distinct roles (Mishima et al., 2014; Schubert et al., 2014; Vlaskamp et al., 2016). In light of these findings, we propose that syntaxin-1A and syntaxin-1B perform different roles in mediating mTORC1-dependent regulation of postsynaptic membrane potential as described below.

Another mTORC1-regulated protein that caught our attention was the ionotropic glutamate receptor NMDA-type subunit 1 (GluN1). GluN1 is the obligate subunit for functional NMDARs (Cull-Candy and Leszkiewicz, 2004; Paoletti, 2011). Interestingly, GluN1 was dramatically elevated with reduced mTORC1 activity, such that we could only detect it in the PSD fraction by MS/MS after administering rapamycin as reported (Niere et al., 2016). GluN1, hence, is an mTORC1-Off protein. Because the mRNAs encoding GluN1 and syntaxins 1A and 1B are in the dendrites, we hypothesize that they are postsynaptically translated in response to changes in mTORC1 activity (Cajigas et al., 2012). Namely, Stx1B is synthesized when mTORC1 is turned on and GluN1 and Stx1A when mTORC1 is turned off.

In this section, we consider our data in the context of what others have demonstrated regarding syntaxins 1A, 1B and NR1. We propose that these proteins maintain the oscillation of mTORC1 between “On” and “Off” states that determines the excitability of the postsynaptic membrane (Figure 1). We are calling this pathway the negative feedback loop. The “active” direction of the negative feedback loop begins when mTORC1 is turned off and ends when mTORC1 is turned on. In this process, repression of mTORC1 activity increases the protein levels of Stx1A and GluN1 in the PSD and resultantly turns mTORC1 on. The “inactive” direction of the negative feedback loop toggles mTORC1 from “On” to “Off” state. In the negative path, Stx1B mediates the switch in mTORC1 activity.

### mTORC1-Dependent Expression of Stx1A and GluN1 May Increase Postsynaptic Membrane Excitability

A recent study demonstrated that postsynaptic Stx1A preferentially associates with NMDARs over α-amino-3-hydroxy-5-methyl-4-isoxazolepropionic acid receptors (AMPARs; Hussain et al., 2016). Earlier studies implicated syntaxins 3 and 4 but not syntaxin-1 in AMPAR exocytosis during LTP (Kennedy et al., 2010; Jurado et al., 2013; Jurado, 2014). In light of these recent findings, we predict that in states where mTORC1 activity is attenuated, the elevated levels of NR1 and syntaxin-1 serve to increase the surface expression of NMDARs at the postsynaptic membrane. Increased NMDAR density can contribute to postsynaptic excitability through their interaction with other voltage-sensitive conductances (e.g., voltage-gated calcium channels and calcium-activated potassium channels; Poolos and Kocsis, 1990; Pongrácz et al., 1992; Schiller et al., 2000; Schiller and Schiller, 2001; Stocker, 2004; Antic et al., 2010; Faber, 2010; Shah et al., 2010). Therefore, turning down mTORC1 activity acts as a signal to increase dendritic membrane excitability.

Completing the active-negative feedback pathway, increased NMDAR surface expression can escalate mTOR activation through calcium-mediated signaling cascade (Tang and Schuman, 2002; Gong et al., 2006; Hoeffer and Klann, 2009). This, in turn, will increase the expression of mTORC1-On proteins, such as Stx1B but decrease the levels of NR1 and Stx1A (Figure 1). We predict that turning on mTORC1 elevates Stx1B postsynaptically and leads to degradation of NMDARs. Consequently, removal of NMDARs will turn off mTORC1. NMDAR activation can lead to its internalization (Vissel et al., 2001; Barria and Malinow, 2002; Nong et al., 2003). Several mechanisms control NMDAR internalization, recycling and degradation (Roche et al., 2001; Prybylowski et al., 2002; Scott et al., 2004). The NMDAR subunits GluN1 and GluN2 contain endocytic sorting domains (Scott et al., 2004). GluN2B contains two motifs and thus GluN2B can be directed either to the recycling or late endosome/lysosomal pathway. GluN1, however, solely contains the motif that directs the receptor to the lysosomal or degradation path. The degradative path of GluN1 is highly interesting as our MS/MS data only detects an increase in GluN1 when mTORC1 activity is attenuated (Table 1, Niere et al., 2016). As a requisite subunit for functional NMDARs, mTORC1-dependent upregulation of NR1 may be critical in maintaining membrane excitability (Cull-Candy and Leszkiewicz, 2004; Paoletti, 2011).

### mTORC1-Dependent Expression of Stx1B May Reduce Postsynaptic Membrane Excitability

Syntaxin-11 has recently been implicated in late endosome-lysosome fusion (Offenhäuser et al., 2011; van der Sluijs et al., 2013). Curiously, syntaxin-11 protein and mRNA are essentially absent in the brain (Valdez et al., 1999). A comparison of different syntaxins reveals that syntaxin-1B and syntaxin-11 have extensive homology (Tang et al., 1998). Thus, we suspect that in the brain, syntaxin-1B may be the functional homolog to syntaxin-11 by tethering NR1 to the lysosomes for degradation when mTORC1 is active.

By sensing calcium levels mediated by NMDARs, mTORC1 accordingly tunes the electrical property of the postsynaptic membrane through a negative feedback mechanism that relies on the protein expression of syntaxins 1A and 1B and NR1 (Table 1 and Figure 1). mTORC1 increases membrane excitability by synchronously increasing Stx1A and decreasing Stx1B to escalate surface expression of NMDARs through elevated NR1 protein levels. However, as mTORC1 gets turned on by increasing calcium concentration through NMDAR entry, mTORC1 simultaneously reduces Stx1A and elevates Stx1B protein levels to inhibit NMDAR trafficking to the membrane. This response can lead to a reduction in mTORC1 activity.

## Local Voltage Sensing of mTORC1 Is Vital for Normal Postsynaptic Function and Requires Dynamic Expression of mTORC1-On and off Proteins

In consideration of our data and published observations, we have attempted to synthesize a mechanism by which mTORC1 can detect changes in local, postsynaptic membrane potential. This proposed mechanism implicates mTORC1 as a voltage sensor that can promote protein synthesis of specific receptors, ion channels, and associated proteins that can keep mTORC1 activity in the optimal range. Interestingly, protein syntheses of several ionotropic receptors, ion channels, and their associated proteins are dependent on mTOR activity (Tang et al., 2002; Cammalleri et al., 2003; Schratt et al., 2004; Raab-Graham et al., 2006; Liao et al., 2007; Gobert et al., 2008; Meyuhas and Kahan, 2015).

Our biochemical, bioinformatics and imaging data have identified molecules that can mediate mTORC1-dependent regulation of the postsynaptic membrane. Emerging data support a role for clustered synaptic plasticity both *in vivo* and *in vitro* (Govindarajan et al., 2006; Losonczy and Magee, 2006; Losonczy et al., 2008; Kleindienst et al., 2011; Makino and Malinow, 2011). However, to test this hypothesis, electrophysiological experiments and/or imaging experiments that evaluate the necessity of Stx1A and Stx1B to shuttle GluN receptors to and from the postsynaptic membrane are critical. The relationship between acute changes in local, postsynaptic mTORC1 activity and surface membrane expression of GluN should also be tested. Work by Makino and Malinow (2011) elegantly demonstrate fluorescent tagged GluA1 receptors enriched in spines that are clustered together upon sensory experience. Perhaps a similar strategy can be utilized for GluN1.

A-type potassium channels have been implicated as a critical factor in determining dendritic branch strength (Losonczy et al., 2008; Makara et al., 2009). The molecular identity underlying the A-type current, however, has been suggested to be K_v_4.2. Interestingly, K_v_4.2 is an mTOR-Off protein (Lee et al., 2011). In light of our data, K_v_1.1 could also serve as a gatekeeper of branch strength and compartmentalization (Figures 2–5; Sosanya et al., 2015b). Determining the contribution of K_v_1.1 to branch strength, however, is more difficult due to its presence in both axons and dendrites when mTOR is off. Genetic tools that block or increase branch-specific dendritic targeting of K_v_1.1 are necessary to adequately test our hypothesis.

How can positive and negative feedback pathways that control mTORC1 activity take place in a single neuron? One way this is achieved is by localizing different populations of mRNAs-RNA binding protein (RNP) complex to specific dendritic branches. For example, we have recently shown that the RNA binding protein HuD favors 1 daughter branch over the other (Sosanya et al., 2015b). Our model suggests that the choice between translating *Stx1A* and/or *Stx1B* mRNA dictates whether a dendrite will utilize a negative or positive feedback pathway. Future studies should include the identification of the RNA binding factors that regulate *Stx1A* and/or *Stx1B* mRNA translation to determine if they are expressed in unique dendritic branches similar to HuD.

Many mTOR-related diseases present mTORC1 activity that is outside the ideal physiological range. The recruitment of a positive feedback may underlie mTORC1-related diseases. For example, excessive repression of the inactive arm would drive the hyperactivity of mTOR and the repression of K_v_1.1, 1.2, β2 and Lgi1 (Figure 1C). This is likely to be the case in animal models of diseases that exhibit neurological disorders that are common in Alzheimer’s disease, autism spectrum disorder, and tuberous sclerosis complex, all disease where patients are reported to suffer from seizures (Swiech et al., 2008; Bateup et al., 2011, 2013; Bové et al., 2011; Hays et al., 2011; Ronesi et al., 2012; Contractor et al., 2015; Tamagnini et al., 2015a,b; Oh et al., 2016). In contrast, extended use of the inactive arm would induce constitutive expression of K_v_1.1, 1.2, β2, and Lgi1 that could lead to dangerously low mTOR activity and diseases with hypoactive mTOR (e.g., Parkinson’s disease, Rett’s syndrome, and major depressive disorder; Karege et al., 2007; Swiech et al., 2008; Taneja et al., 2009; Bové et al., 2011; Jernigan et al., 2011; Ricciardi et al., 2011; Chandran et al., 2013; Figure 1B). Considering the presence of mTORC1 in the postsynaptic region and its ability to regulate the levels of ion channels, ionotropic receptors, and their associated proteins that determine conductance and trafficking, it is germane to define the link between dysregulated mTORC1 activity and abnormal membrane excitability. While it may be that the proposed positive and negative feedback mechanisms governing mTORC1-dependent regulation of membrane potential may be simple as presented, consideration and refinement of this hypothesis is an important step that will have broad implications not only in neurological disorders but in other fields that investigate excitable membranes.

## Ethics Statement

This study was carried out in accordance with the recommendations of National Institutes of Health’s Guide for the Care and Use of Laboratory Animals. The protocol was approved by the University of Texas Institutional Animal Care and Use Committee.

## Author Contributions

FN and KFR-G designed the experiments and wrote the article. FN performed the experiments and analyzed the data.

## Conflict of Interest Statement

The authors declare that the research was conducted in the absence of any commercial or financial relationships that could be construed as a potential conflict of interest.
